# Flavanone-Based Fluorophores with Aggregation-Induced Emission Enhancement Characteristics for Mitochondria-Imaging and Zebrafish-Imaging

**DOI:** 10.3390/molecules25143298

**Published:** 2020-07-21

**Authors:** Na Li, Liyan Liu, Huiqing Luo, Huaqiao Wang, Depo Yang, Feng He

**Affiliations:** 1 School of Pharmaceutical Science, Sun Yat-sen University, Guangzhou 510006, China; lina49@mail2.sysu.edu.cn (N.L.); liuly37@mail2.sysu.edu.cn (L.L.); luohq8@mail2.sysu.edu.cn (H.L.); lssydp@mail.sysu.edu.cn (D.Y.); 2 Department of Anatomy and Neurobiology, Zhongshan School of Medicine, Sun Yat-sen University, Guangzhou 510006, China; wanghq@mail.sysu.edu.cn

**Keywords:** AIEE, flavanone, mitochondria, zebrafish

## Abstract

Fluorophores with aggregation-induced emission enhancement (AIEE) characteristics applied in bioimaging have attracted more and more attention in recent years. In this work, a series of flavanone compounds with AIEE characteristics was developed and applied to fluorescence imaging of mitochondria and zebrafish. The compounds were readily prepared by the thermal dehydration of chalcone that was obtained by the reaction of *o*-hydroxyacetophenone and benzaldehyde. Two of these compounds showed significant AIEE characteristics by fluorescence performance experiments, including optical spectra, fluorescence spectra, fluorescence quantum yield (φ_F_), fluorescence lifetime, and scanning electron microscopy (SEM). Compared with traditional organic fluorescent dyes, these compounds have high fluorescence emission and high fluorescence quantum yield in solid or aggregated state, which overcomes the shortcoming of aggregation-caused quenching (ACQ). More importantly, the two compounds exhibited low cytotoxicity and good cytocompatibility in A549 lung cells at the experimental concentration range and they specifically targeted mitochondria, which make it of great potential use in mitochondria labeling. In addition, they were embryonic membrane permeable and had different affinities for different tissues and organs of zebrafish, but mainly distributed in the digestive system, providing a basis for the application of such compounds in bioimaging. These AIEE compounds with superior properties could be of great potential use in mitochondria imaging and other in vivo studies.

## 1. Introduction

Organic fluorescent dyes have been widely used in bioimaging in recent years because of their excellent optical and biological properties, such as high fluorescence quantum yield, good biocompatibility and optical stability [1,2,3,4,5,6]. It is very valued for biological application but traditional fluorescent dyes have an effect called aggregation-caused quenching (ACQ). Traditional fluorescent dyes with ACQ characteristics emit strong fluorescence in monodisperse or dilute solutions, but emit weak fluorescence while molecules are aggregated at high concentrations or in solid state, so the application of these dyes is limited [7]. The main reason for this phenomenon is that intermolecular π-π stacking of the aromatic rings connected to the fluorophores causes molecules in excited state return to ground state in a non-radiative manner [8]. In 2001, Tang’s group found aggregation-induced emission enhancement (AIEE) phenomenon which was completely opposite to ACQ [9]. Molecules with AIEE characteristics do not emit fluorescence or show very weak fluorescence in monodisperse or dilute solutions, but emit strongly at high concentrations or in solid state. Then Tang’s group synthesized a series of compounds with AIEE characteristics, such as silicon heterocyclic polyene, diphenyldibenzofulvene (DPDBF) derivatives, pyran derivatives, polyphenyl substituted alkenes (such as butene, cyclobutene, and polyphenylene) [10,11,12,13,14]. There are two main reasons for AIEE phenomenon, namely restriction of intramolecular rotations (RIR) and restriction of intramolecular vibrations (RIV) [15,16]. Since the first report of AIEE molecules, these molecules have been widely studied and applied as chemical probes, bioprobes, and stimulus response probes, which provided a new way for organic luminescent materials to be applied in solid devices and high concentration conditions [17,18,19,20].

Flavonoids are secondary metabolites in plants and are widely found in nature. Flavonoids have lots of pharmacological activities, such as anti-tumor, anti-viral, anti-bacterial, hypoglycemic, and so on [21,22,23]. There are cross-conjugated systems in the molecular structure of flavonids, so some of them can emit fluorescence, like anthocyanin. Such molecules have strong fluorescence intensity, strong fluorescence stability, various fluorescent colors that compounds with different structures and different pH conditions have different fluorescent colors [24]. In our previous study, we found a series of flavonoid derivatives with AIEE characteristics, which showed good cellular uptake and low cytotoxicity at the experimental concentrations range. Moreover, they have good specificity for mitochondrial targeting and mitochondrial morphological change tracking [25]. Furthermore, flavanones also belong to flavonoids, which have one less double bond than flavonoids and they are also called dihydroflavones. So in this work, a series of flavanones were synthesized from the thermal dehydration of chalcone obtained from the reaction of acetophenone and benzaldehyde containing different substituents. As a result, compounds **1** and **2** exhibited typical AIEE characteristics and good biocompatibility, making it of great potential use to bioimaging in living A549 cells and vertebrate animal zebrafish.

## 2. Results and Discussion

### 2.1. Optical Properties

**Figure 1:**
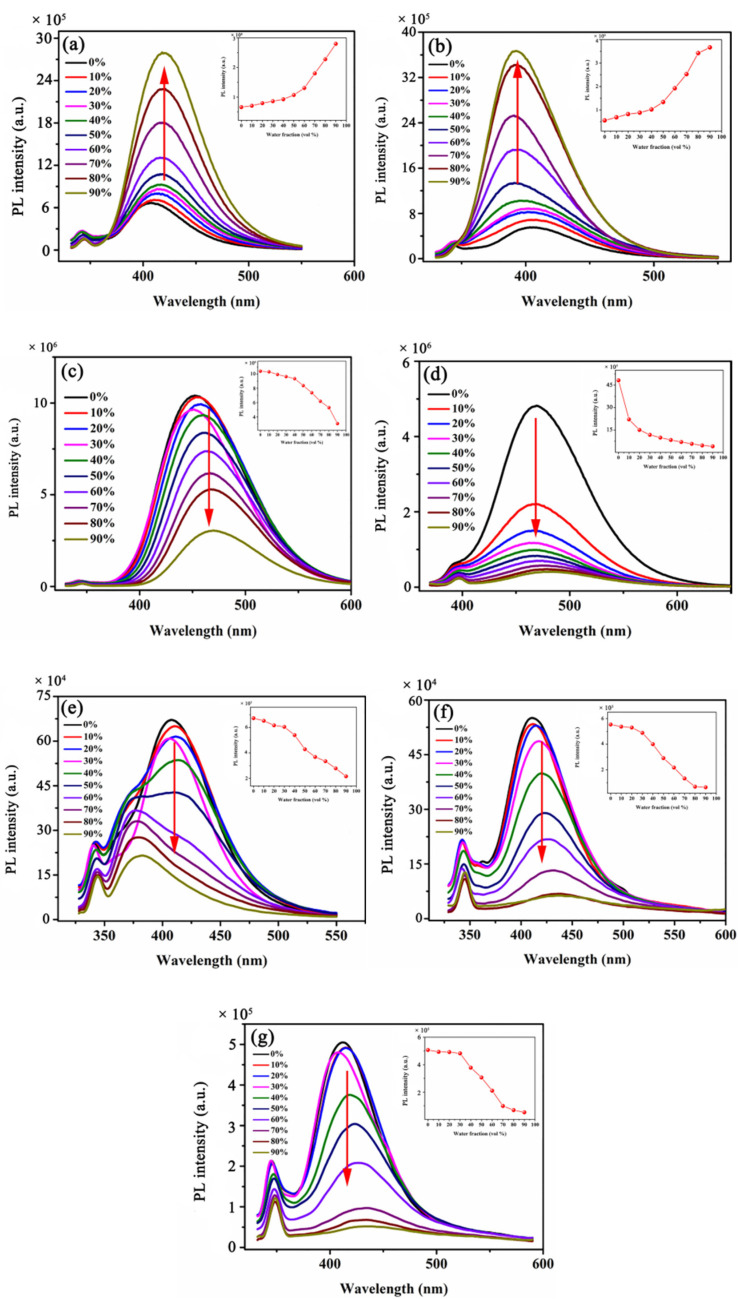
Fluorescence spectra of compounds in CH_3_OH/H_2_O mixed solutions (c = 2.07 × 10^−5^ M) with different water fractions (0–90%) (**a**) **1**, (**b**) **2**, (**c**) **3**, (**d**) **4**, (**e**) **5**, (**f**) **6**, (**g**) **7**.

The optical properties of compounds **1**–**7** in mixed solutions of CH_3_OH/H_2_O with different ratios were investigated by UV-vis spectra and PL spectra. In Figure 1a,b, the PL intensities were weak in pure CH_3_OH solution. As the water fractions increased from 0 to 90%, the PL intensity gradually increased, and it always showed an upward trend. When the water fraction was 90%, the PL intensity reached the maximum. In the tendency of the maximum fluorescence intensity with different water fractions, the maximum of PL intensity also showed a significant upward trend with the increase of water fractions. These results indicated that compounds **1** and **2** have typical AIEE characteristics [26]. However, in Figure 1c–g, the PL intensities of these compounds showed a change rule opposite to that of compounds **1** and **2**. When the water fraction was 0%, the PL intensities of compounds were the strongest, and the peak value in the spectra was the largest. When the water fraction changed from 0 to 90%, the PL intensity gradually decreased and reached the minimum when the water fraction was 90%. In the trend diagrams of the maximum PL intensity, as the water fraction increased, the maximum of the PL intensity also showed a significant downward trend, and the fluorescence gradually quenched, indicating that compounds **3**–**7** are all compounds with ACQ characteristics [26].

From the results of the PL spectra, flavanone derivatives **1** and **2** exhibited significant AIEE characteristics. To further study the AIEE characteristics of the two compounds, UV-vis absorption spectra of compounds **1** and **2** with water fractions changing from 0 to 90% were measured.

**Figure 2:**
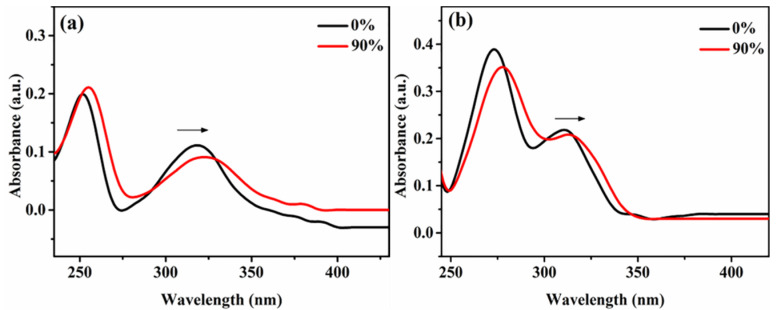
UV-vis absorption spectra of compounds mixed solution with water fraction changing from 0 to 90% (**a**) **1**, (**b**) **2**.

As can be seen in Figure 2, the pure CH_3_OH solutions of compounds **1** and **2** showed absorption peaks at 317 and 311 nm, respectively. In Figure 2a, when the water fractions changed from 0 to 90%, there was significant red shift. Figure 2b also showed the same phenomenon. The above results suggested that the solutions of compounds **1** and **2** with 90% water fraction may form a new aggregate capable to promote fluorescence enhancement [27], thereby increasing the PL intensity of the mixed solutions, which is consistent with the results of the above fluorescence spectra. As the water fractions increases, the new aggregates in the solution become more and more, so the PL intensity becomes stronger and stronger, which is consistent with the characteristic of AIEE compounds.

In order to study in more detail the fluorescence properties of compounds **1** and **2**, the fluorescence quantum yields (φ_F_) and fluorescence lifetime of the two compounds with different water fractions were measured. In Table 1, the fluorescence quantum yields of compound **1** in CH_3_OH, CH_3_OH/H_2_O (5:5 *v*:*v*) and CH_3_OH/H_2_O (1:9 *v*:*v*) were 0.02, 0.07, and 0.13, the fluorescence lifetime was 2.12, 2.26, and 2.98 ns. It was indicated that the tendency of fluorescence quantum yield and fluorescence lifetime were consistent, both of which reach the maximum when the water fraction was 90%, which is also in accordance with the PL spectra of compound **1**. Compound **2** also showed the same characteristic. The fluorescence quantum yield and fluorescence lifetime of compound **2** reached the maximum when the water fraction was 90%, and its change tendency was consistent with the change in the PL spectra. It is also confirmed that compounds **1** and **2** are typical AIEE compounds.

**Figure 3:**
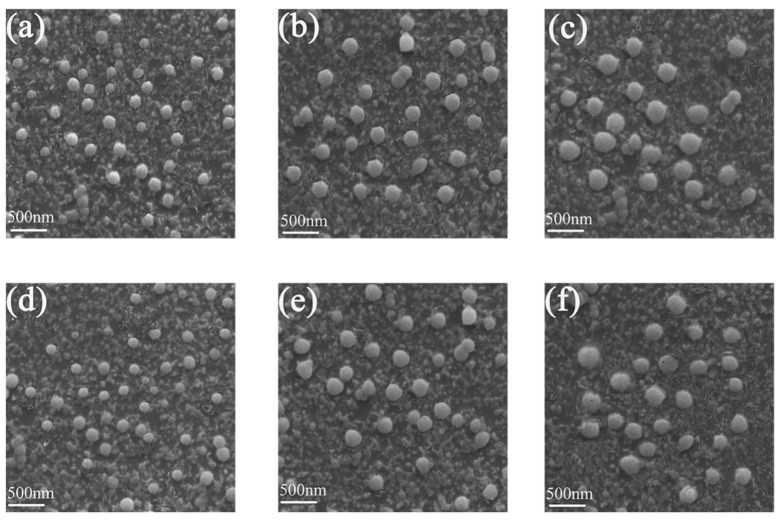
SEM images in the mixed solutions of CH_3_OH/H_2_O (5:5 *v*:*v*) (**a**) **1**, (**d**) **2**. SEM images in the mixed solutions of CH_3_OH/H_2_O (2:8 *v*:*v*) (**b**) **1**, (**e**) **2**. SEM images in the mixed solutions of CH_3_OH/H_2_O (1:9 *v*:*v*) (**c**) **1**, (**f**) **2**.

For further confirming the reason for AIEE characteristics of compounds **1** and **2**, the particles of the compounds with different water fractions were observed by SEM. Figure 3a–c are the images of SEM of compound **1** in the CH_3_OH/H_2_O (5:5, 2:8, 1:9 *v*:*v*) mixed solutions. The particles appeared as uniformly dispersed spherical particles, and the spherical particles were small when the water fraction was 50%. The diameter of spherical particles in the solution gradually increased as the water fractions increased. When the water fraction was 90%, the spherical particles were the biggest. Figure 3d–f shows the SEM images of compound **2** in the CH_3_OH/H_2_O (5:5, 2:8, 1:9 *v*:*v*) mixed solutions. The change of particles were in accordance with that of compound **1**. The SEM images indicated that the diameter of the particles increased when the water fraction changed from 50 to 90%. By comparing with the PL spectra, it can be inferred that the increase of the diameter of the particles are advantageous for enhancing the PL intensity.

**Figure 4:**
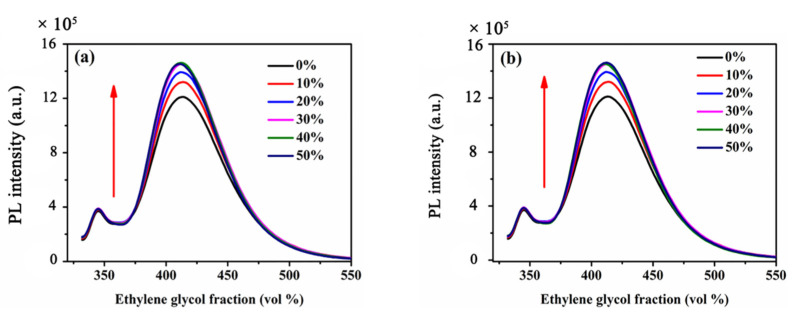
Fluorescence spectra of compounds in the mixed solutions of CH_3_OH/EG (*v*:*v*) (**a**) **1**, (**b**) **2**.

In our previous study, we found that RIR should be the main reason for the AIEE phenomenon in flavonoids derivatives [25]. Therefore, in order to study the reason of AIEE characteristics of flavanone-based compounds **1** and **2**, the viscosities of compounds **1** and **2** solutions were increased by adding different ratios of ethylene glycol to the solutions, and then PL intensities of the solutions were measured. In Figure 4a, when the EG fractions increased from 0 to 50%, the viscosities of solutions gradually increased, and the PL intensities of compound **1** in the CH_3_OH/EG also gradually increased. Figure 4b also showed the same rule change. With increasing viscosity, the rotation of the molecules in the mixed solutions was limited and fluorescence enhanced. It can be speculated that the reason of AIEE in compounds **1** and **2** should also be RIR, which is consistent with flavonoids.

### 2.2. Cell Imaging

**Figure 5:**
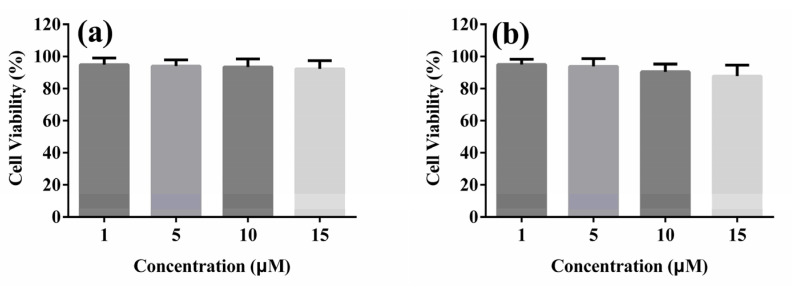
Cell viability of A549 cells treated with different concentrations (**a**) **1**, (**b**) **2** for 24 h.

To investigate these AIEE compounds whether can be further applicated in biology, cytotoxicity of compounds **1** and **2** in A549 cells was tested by using MTT assay. A549 cells were incubated with different concentrations (1, 5, 10 and 15 μM) of compounds **1** and **2** for 24 h. The results are shown in Figure 5, which concluded that compounds **1** and **2** showed low cytotoxicity to A549 cells within 24 h. Cell viability exceeded 80% at each concentrations, indicating that compounds **1** and **2** have good cytocompatibility in the experimental concentration range.

**Figure 6:**
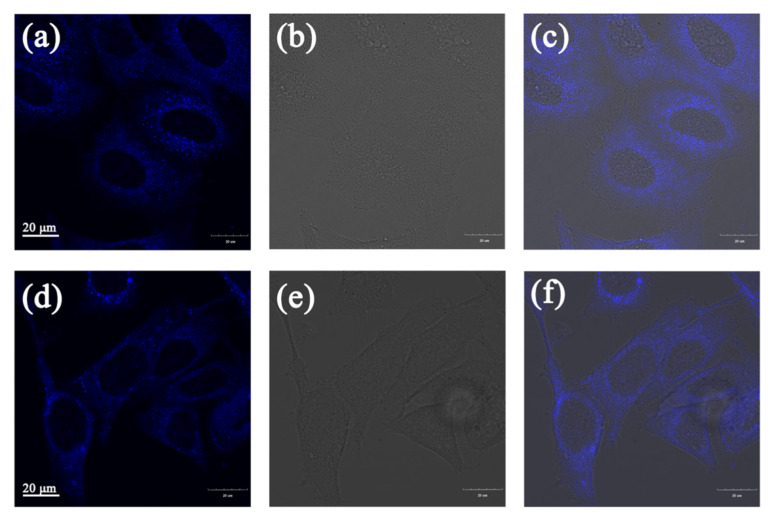
(**a**) The fluorescence image of compound **1** in A549 cells. (**b**) The bright-field image. (**c**) The merge image of (**a**) and (**b**). (**d**) The fluorescence image of compound **2** in A549 cells. (**e**) The bright-field image. (**f**) The merge image of (**e**) and (**f**). The excitation wavelength was 359 nm. The collected wavelength range was 430–480 nm.

In the previous section, the cytotoxicity of compounds **1** and **2** was investigated by MTT assay, and it was found that both the two compounds have low cytotoxicity, indicating that they can be further applied in living cells. Therefore, cellular uptake experiments were performed with A549 cells. A549 cells were incubated with 10 μM of compounds **1** and **2** in an incubator for 30 min, and then washed three times with PBS for confocal imaging. In Figure 6, bright blue fluorescence was observed, which suggested that compounds **1** and **2** can be taken up by A549 cells and exhibit good cytocompatibility. Most of the blue fluorescence was observed to accumulate in the cytoplasm of A549 cells. These compounds aggregated in the cytoplasm of the cells and emitted strong fluorescence, showing good cellular uptake, indicating that compounds **1** and **2** can be applied to bioimaging.

**Figure 7:**
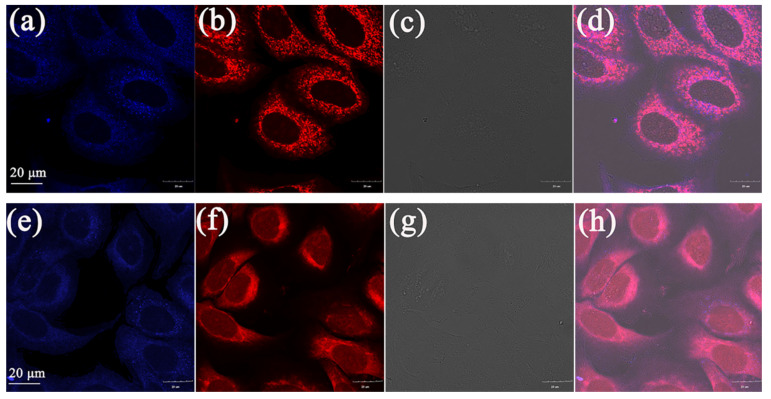
Co-localized images of A549 cells incubated with (**a**) **1**, (**e**) **2** (10 μM) for 30 min and Mito Tracker Red (200 nM) (**b**), (**f**) for 15 min. The bright-field image of A549 cells stain with (**c**) **1**, (**g**) **2**. The merged image stained with (**d**) **1**, (**h**) **2**. The excitation wavelength of compound **1** and **2** was 359 nm and the collected wavelength range was 430–480 nm. The excitation wavelength of Mito Traker Red was 578 nm and the collected wavelength range was 570–620 nm.

**Figure 8:**
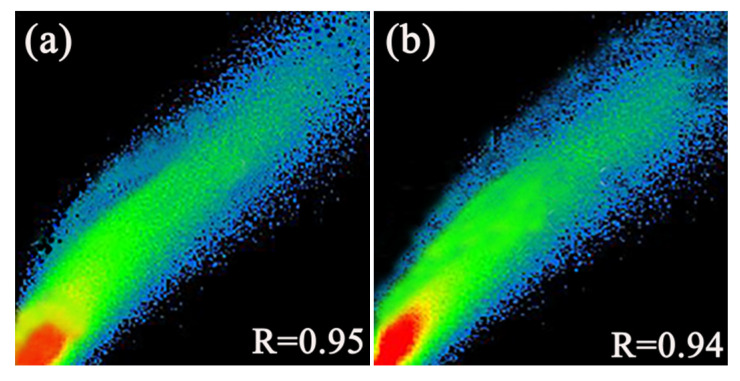
(**a**) R value of compound **1** and Mito Tracker Red (**b**) R value of compound **2** and Mito Tracker Red.

In order to investigate whether they accumulate in mitochondria like flavonoid AIEE compounds, mitochondrial co-localization experiments were performed. Mito Tracker Red (200 nM) and compounds (10 μM) were stained together and then observed. Both compounds **1** and **2** showed bright blue fluorescence in Figure 7a,e, and the mitochondria in the cytoplasm showed red fluorescence in Figure 7b,f. Moreover, the stained site of compounds **1** and **2** and that of Mito Tracker Red are highly coincident in Figure 7d,h. In order to accurately analyze the coincidence, the corresponding R values (Pearson correlation coefficient, the coefficient indicating the degree of coincidence, range +1 to −1) were measured, and the results are shown in Figure 8. The coefficients were 0.95 and 0.94, respectively, indicating compounds **1** and **2** can specifically aggregate in mitochondria and label mitochondria, suggesting that they can be applied in mitochondrial imaging.

### 2.3. Zebrafish Imaging

**Figure 9:**
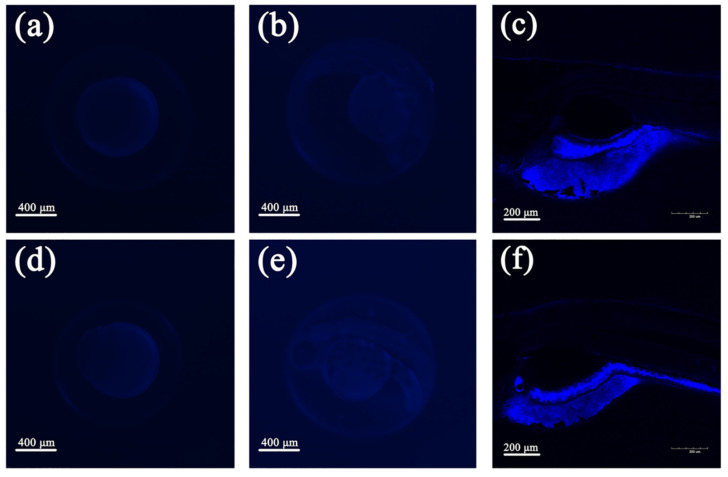
Fluorescence images of zebrafish embryos after soaking in the mixed solutions (15 μM) of compounds **1** (upper) and **2** (lower) for 3 h at time points: (**a**,**d**) 9, (**b**,**e**) 48, (**c**,**f**) 120 hpf. The excitation wavelength was 359 nm. The collected wavelength range was 430–480 nm.

Zebrafish is a simple but important biological laboratory animal, which is widely used in pharmaceutical research. Zebrafish is in vitro fertilized and has up to 87% genetic similarity with human. Importantly, the embryo of zebrafish is almost transparent and the internal tissues and organs are observed [28,29,30,31]. The above results indicated that these flavanones derivatives have a good application prospect in living cell imaging, so for further studies the application of such compounds in bioimaging, zebrafish was selected as the research object. In Figure 9, after administration of 6 hpf (hours post fertilization) zebrafish embryos, the yolk of the embryo showed bright blue fluorescence, indicating that the compounds passed through the embryo membrane into the embryo by simple infiltration. After 48 h, the blue fluorescence also mainly concentrated in the yolk sac. After 5 days, the blue fluorescence also mainly concentrated in the yolk sac and intestinal tract, indicating that the compounds have different affinities for different tissues and organs of zebrafish, and mainly concentrated in the digestive system.

## 3. Materials and Methods

### 3.1. Materials and Instruments

2-hydroxy-5-methoxya-cetophenone, 2-hydroxyacetophenone, 2,4-dihydroxyacetophenone, 2-hydroxy-5-methoxyacetophenone, 2-hydroxy-4-methoxyacetophenone, benzaldehyde, pyridine, 2,5-dihydroxyacetophenone, hydrochloric acid, methanol solution, sodium hydroxide, concentrated sulfuric acid, dichloromethane, dimethylsulfoxide (DMSO), petroleum ether, and ethyl acetate were purchased from Macklin, Sigma-Aldrich and Aladdin (Shanghai, China). Liquiritigenin (compound **5**), naringenin (compound **6**), and hesperetin (compound **7**) were purchased from Aladdin (Shanghai, China). The human lung cancer cell line A549 was obtained from laboratory animal center of Sun Yat-sen University. Rosewell Park Memorial Institute 1640 medium (RPMI-1640), fetal bovine serum (FBS), 0.25% trypsin solution, penicillin-streptomycin solution, agarose, phosphate buffered saline (PBS), 3-(4,5-dimethyl-2-thiazolyl)-2,5-diphenyl-2-H-tetrazolium bromide (MTT), and Mitotracker Red assay kit (MT) were purchased from Thermo-Fisher Biochemical Products (Beijing, China). All the regents and analytical grade solvents were purchased from commercial suppliers and used without further purification unless indicated. 1H-NMR (400 MHz) and 13C-NMR (101 MHz) spectra were obtained on an Avance Ⅲ 400 MHz spectrometer (Bruker, Karlsruhe, Germany) in CDCl3. The compounds produced in reaction were checked by analytical thin layer chromatography (TLC). The high-resolution mass spectra were measured on a LCMS-IT-TOF mass spectrometer (Shimazu, Kyoto, Japan). Photoluminescence (PL) spectra and absolute PL quantum yields were collected from a FLS 920 spectrophotometer (Edinburgh Instruments, Edinburgh, UK). The fluorescence lifetime was made on a FLS 980 spectrometer (Edinburgh Instruments, Edinburgh, UK). Ultraviolet (UV) absorption spectra were collected from a UV-2600 spectrometer (Shimadzu, Kyoto, Japan). Scanning electron microscope (SEM) images were obtained from a Zeiss Merlin emission scanning electron microscope (Zeiss Co., Oberkochen, Germany). Fluorescent images were obtained from a OLYMPUS FV3000 laser scanning confocal microscope (Zeiss, Oberkochen, Germany). Cell viability was analyzed by a Flex Station 3 microplate read (Molecular Devices, silicon vally, CA, USA). Fluorescent images of zebrafish embryo were performed on a EVOS FL Auto imaging system (Life Technologies, Carlsbad, CA, USA).

### 3.2. Synthesis of Flavanone Derivatives

**Figure 10:**
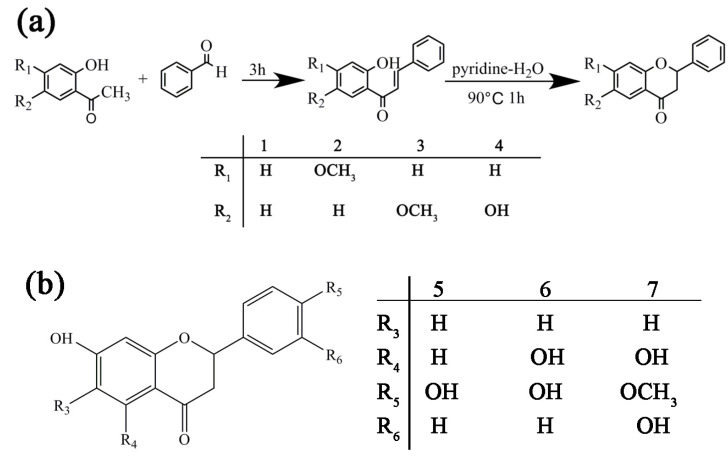
(**a**) The reaction route of flavanone derivatives **1**–**4**. (**b**) Chemical structure of compounds **5**–**7**.

The synthesis route of flavanone derivatives is shown in Figure 10. The synthesis includes two steps. In the first step, *o*-hydroxyacetophenone containing different substituents and benzaldehyde react with sodium hydroxide and ethanol to produce chalcone containing different substituents. The second step is the thermal dehydration of chalcone in pyridine and water to produce flavanones. Characterization of compounds **1**–**4** can be seen in the (Supplementary Materials Figures S1–S4).

#### 3.2.1. Synthesis of Compound **1**

Sodium hydroxide (1.00 g) and water (5 mL) were added into a 100 mL beaker, stirred to dissolve, and then added dropwise 2-hydroxyacetophenone (1.4 mL) and ethanol (2.5 mL). The mixed solution of ethanol (1.0 mL) and benzaldehyde (0.9 mL) was slowly added to the beaker under stirring. Then the mixture was stirred at room temperature for 3 h. When the mixed solution gradually turned to be orange and accompanied by the formation of solid precipitation, the system was placed in an ice-water mixture for refrigeration. Subsequently, 5% hydrochloric acid was added dropwise to the beaker until the system was weakly acidic, filtered, and then the solid was recrystallized with 95% ethanol. After suction filtration and drying, 2-hydroxychalcone was obtained.

2-hydroxychalcone and pyridine-water (4:6, 180 mL) solution were added into a 250 mL round bottom flask equipped with a reflux condenser, and stirred at 90 °C for 1 h. Next the water in the solution was extracted with dichloromethane, and then the residual solution was treated with a rotary evaporator. The obtained solid was purified by silica gel column chromatography (petroleum ether:ethyl acetate = 50:1) to get the target compound flavanone.

#### 3.2.2. Synthesis of Compound **2**

Sodium hydroxide (1.00 g) and water (5 mL) were added into a 100 mL beaker and stirred to dissolve, then 2-hydroxy-4-methoxyacetophenone (1.95 g) and ethanol (2.5 mL) were added. The latter method is the same as 3.2.1, finally the target product 7-methoxyflavanone was obtained.

#### 3.2.3. Synthesis of Compound **3**

Sodium hydroxide (1.00 g) and water (5 mL) were added into a 100 mL beaker and stirred to dissolve, then 2-hydroxy-5-methoxyactophenone (1.95 g) and ethanol (2.5 mL) were added. The latter method is the same as 3.2.1, and finally the target compound 6-methoxyflavanone was obtained.

#### 3.2.4. Synthesis of Compound **4**

Sodium hydroxide (1.00 g) and water (5 mL) were added into a 100 mL beaker and stirred to dissolve. Then 2,5-dihydroxyacetophenone (1.75 g) and ethanol (2.5 mL) were added. The latter method is the same as 3.2.1, and finally the target product 6-hydroxyflavanone was obtained.

*Flavanone* (**1**), Yield: 63%; slight yellow solid, mp.: 75–78 °C; 1H-NMR (400 MHz, CDCl3) δ 7.88 (s, 1H), 7.39 (d, *J* = 17.6 Hz, 6H), 7.00 (s, 2H), 5.44 (s, 1H), 3.03 (s, 1H), 2.85 (s, 1H); 13C-NMR (101 MHz, CDCl3) δ 190.95 (s), 162.60 (s), 160.55 (s), 137.73 (s), 127.84 (s), 127.84 (s), 127.76 (s), 126.05 (s), 125.13 (s), 125.13 (s), 120.61 (s), 119.93 (s), 117.12 (s), 78.59 (s), 43.66 (s); IR-KBr: 3060, 3000, 2900, 2850, 1690, 1600, 1580, 1460, 1410, 1370, 1300, 1230, 1110, 1030 cm^−1^; HRMS (ESI-MS) *m/z* calcd for C15H12O2 [M + H]+: 225.0871; found: 225.0915.

*7-Methoxyflavanone* (**2**), Yield: 52%; slight yellow solid, mp.: 89–91 °C; 1H-NMR (400 MHz, CDCl3) δ 7.80 (d, *J* = 8.8 Hz, 1H), 7.38 (dd, *J* = 10.5, 4.0 Hz, 5H), 6.55 (s, 1H), 6.43 (d, *J* = 2.4 Hz, 1H), 5.40 (dd, *J* = 13.3, 2.9 Hz, 1H), 3.76 (s, 3H), 2.97 (dd, *J* = 16.9, 13.3 Hz, 1H), 2.82–2.69 (m, 1H); 13C-NMR (101 MHz, CDCl3) δ 189.58 (s), 165.17 (s), 162.50 (s), 137.75 (s), 127.82 (s),127.82 (s), 127.74 (s), 125.14 (s), 125.14 (s), 125.14 (s), 113.79 (s), 109.26 (s), 99.88 (s), 78.98 (s), 54.63 (s), 43.30 (s); IR-KBr: 3080, 3020, 2970, 2940, 2830, 1670, 1610, 1570, 1490, 1440, 1380, 1350, 1220, 1120, 1020 cm^−1^; HRMS (ESI-MS) *m/z* calcd for C16H14O3 [M + H]+: 255.0976; found: 255.1022.

*6-Methoxyflavanone* (**3**), Yield: 55%; slight yellow solid, mp.: 141–143 °C; 1H-NMR (400 MHz, CDCl3) δ 7.80 (d, *J* = 8.8 Hz, 1H), 7.38 (dd, *J* = 10.5, 4.0 Hz, 5H), 6.55 (s, 1H), 6.43 (d, *J* = 2.4 Hz, 1H), 5.40 (dd, *J* = 13.3, 2.9 Hz, 1H), 3.76 (s, 3H), 2.97 (dd, *J* = 16.9, 13.3 Hz, 1H), 2.82–2.69 (m, 1H); 13C-NMR (101 MHz, CDCl3) δ 191.06 (s), 155.25 (s), 153.20 (s), 137.81 (s), 127.81 (s), 127.81 (s), 127.71 (s), 125.12 (s), 125.12 (s), 124.39 (s), 119.72 (s), 118.42 (s), 106.30 (s), 78.69 (s), 54.81 (s), 43.51 (s); IR-KBr: 3070, 3000, 2960, 2930, 2830, 1670, 1610, 1580, 1480, 1430, 1380, 1350, 1220, 1120, 1030 cm^−1^; HRMS (ESI-MS) *m/z* calcd for C16H14O3 [M + H]+: 255.0976; found: 255.1022.

*6-Hydroxyflavanone* (**4**), Yield: 50%; slight yellow solid, mp.: 220–222 °C; 1H-NMR (400 MHz, CDCl3) δ 7.44–7.30 (m, 4H), 7.27 (d, *J* = 3.1 Hz, 1H), 7.01 (dd, *J* = 7.3, 4.7 Hz, 2H), 6.91 (d, *J* = 8.9 Hz, 1H), 5.37 (d, *J* = 13.4 Hz, 1H), 3.00 (dd, *J* = 17.0, 13.4 Hz, 1H), 2.80 (dd, *J* = 18.7, 4.6 Hz, 1H), 2.29 (s, 1H); 13C-NMR (101 MHz, DMSO) δ 192.19(s), 154.86(s), 152.09(s), 139.68(s), 128.97(s), 128.97(s), 128.86(s), 126.95(s), 126.95(s), 124.93(s), 121.28(s), 119.37(s), 110.43(s), 79.23(s), 44.17(s). IR-KBr: 3230, 3090, 2960, 2890, 2810, 1660, 1620, 1580, 1500, 1470, 1380, 1320, 1220, 1130, 1060 cm^−1^; HRMS (ESI-MS) *m/z* calcd for C15H12O3 [M + H]+: 241.0820; found: 241.0864.

### 3.3. Preparation for UV-Vis Spectra, PL Spectra, and SEM Measurements

#### 3.3.1. Preparation for UV-Vis Spectra and PL Spectra

Compounds **1**–**7** were accurately weighed: 0.47, 0.53, 0.53, 0.50, 0.54, 0.63, 0.63 mg. Then they were respectively configured as a series of CH_3_OH/H_2_O mixed solutions with different water fractions (f_w_ = 0, 0.1, 0.2, 0.3, 0.4, 0.5, 0.6, 0.7, 0.8, 0.9). The concentration of the final mixed solution was 2.07 × 10^−5^ M. The mixed solution was sonicated for 10 min, then the UV-vis absorption spectra was measured at room temperature and the PL spectra was measured with the excitation wavelengths 312, 310, 310, 350, 315, 309, 312 nm, respectively. The preparation of the compounds for fluorescence lifetime and the fluorescence quantum yield (φ_F_) measurements was same as above.

#### 3.3.2. Preparation for SEM Measurements

Compounds **1** and **2** in CH_3_OH/H_2_O (5:5, 2:8, 1:9, *v*:*v*) mixtures were prepared. After sonication for 10 min, they were added dropwise to silicon wafers respectively, and the silicon wafers were placed at room temperature for 24 h to be naturally evaporated, and then measured by scanning electron microscope.

### 3.4. Preparation for Ethylene Glycol (EG) Measurements

Compounds **1** and **2** were prepared into methanol solutions with a concentration of 2.07 × 10^−4^ M. The solutions (500 μL) were added into 5 mL volumetric flasks and different ratios of ethylene glycol and methanol were added to configure EG/CH_3_OH mixed solutions with different EG fractions (f_w_ = 0, 0.1, 0.2, 0.3, 0.4, 0.5). The concentration of the final mixed solution was maintained at 2.07 × 10^−5^ M. The mixed solutions were sonicated for 10 min before PL spectra measurement.

### 3.5. Cell Culture

A549 cells were cultured in RPMI-1640 culture medium with 10% fetal bovine serum (FBS), 10 mg/mL streptomycin, and 10 mg/mL penicillin in a cell culture incubator with 5% CO_2_ at 37 °C.

### 3.6. MTT Assay

A549 cells were seeded in 96-well plates, and the density was 9 × 10^3^ per well. The 96-well plates were placed in an incubator for 24 h. Then the prepared solutions of compounds **1** and **2** with different concentrations (1, 5, 10, 15 μM) were added respectively and then placed in the incubator. After 24 h, MTT solution (20 μL, 0.5%) was added to each well, and cells were cultured for another 4 h in the incubator. After 4 h, the medium in the wells was carefully removed. Then 150 μL DMSO solution was added to each well, and the 96-well plates were placed on a shaker at low speed for 10 min. After the crystals were sufficiently dissolved, absorbance of plates was measured at 490 nm.

### 3.7. Cell Imaging

A549 cells were seeded in glass bottom dishes with a density of 1 × 10^5^ per dish and incubated in a cell culture incubator for 24 h. Flavanone derivatives solutions (1000 μL, 10 μM) were added respectively and cultured in the incubator for 30 min. Then medium in the dishes was removed, and the dishes were washed three times with PBS. Then the dishes were observed by FV3000 laser scanning confocal microscope under a 60-fold oil immersion lens. The method of mitochondrial colocalization imaging was same as above. A549 cells were seeded in glass bottom dishes and cultured for 24 h, then flavanone derivatives (10 μM) and Mito Tracker Red (MT) (200 nM) were added to the dishes. After incubating for 30 min, the medium was removed and the dishes were washed three times with PBS. Finally, the dishes were observed by FV3000 laser scanning confocal microscopy under a 60-fold oil immersion lens.

### 3.8. Zebrafish Imaging

Zebrafish (wild type AB line) were obtained from Zebrafish Resource Center (Core Lab Plat for Medical Science, Zhongshan School of Medicine, Sun Yat-sen University). All the procedures were carried out according to the Institutional Ethical Guidelines for Animal Experiment. The ethics approval number is SYSU-IACUC-2020-B0659. Zebrafish were seeded under a constant 14 h on/10 h off light cycle at 28.5 °C. Before fluorescence imaging, 6 hpf zebrafish embryos were randomly divided into two groups and the mixed solutions of compounds **1** and **2** (15 μM) were added. After 3 h, the embryos were washed three times with E3 medium and observed by EVOS FL Auto imaging system. Then the embryos were continuously cultured in an incubator at 28.5 °C and observed after 48 h by EVOS FL Auto imaging system, observed after 120 h by FV3000 laser scanning confocal microscopy under a 10-fold lens.

## 4. Conclusions

In summary, flavanone derivatives **1**–**4** were successfully designed and synthesized. Compounds **1** and **2** showed typical AIEE characteristics and the reason has been speculated that is same as that of flavonoids, which is caused by RIR. Moreover, the two compounds have low cytotoxicity, good cytocompatibility and specifically target to mitochondria of A549 lung cells at the experimental concentration range, making it of great potential use in living cell imaging and mitochondrial imaging. The fluorescence images of these compounds in zebrafish suggested that such flavanone-based AIEE compounds can be applied for further in vivo imaging of zebrafish. Finding more natural compounds with AIEE characteristics and their applications in bioimaging are in progress in our laboratory.

## Figures and Tables

**Table 1:** The fluorescence quantum yield and fluorescence lifetime of compounds **1** and **2**.

Compound	Solvents	Quantum Yields (φ_F_)	Time/ns
	CH_3_OH	0.02	2.12
**1**	CH_3_OH/H_2_O (5:5)	0.07	2.26
	CH_3_OH/H_2_O (1:9)	0.13	2.98
	CH_3_OH	0.10	2.67
**2**	CH_3_OH/H_2_O (5:5)	0.17	3.16
	CH_3_OH/H_2_O (1:9)	0.21	3.24

## Supplementary Materials

The following are available online. Figure S1: 1H, 13C NMR spectra and ESI-MS analysis for compound 1. Figure S2: 1H, 13C NMR spectra and ESI-MS analysis for compound 2. Figure S3: 1H, 13C NMR spectra and ESI-MS analysis for compound 3. Figure S4: 1H, 13C NMR spectra and ESI-MS analysis for compound **4**.
