# Supplementary material for: Flavanone-Based Fluorophores with Aggregation-Induced Emission Enhancement Characteristics for Mitochondria-Imaging and Zebrafish-Imaging
Source: Molecules. 2020 Jul 21;25(14):3298. doi: 10.3390/molecules25143298 (PMC7397278; doi:10.3390/molecules25143298)
Supplement: Supplementary file 1 [file molecules-25-03298-s001.pdf]

Supplementary Information

Flavanone-Based Fluorophores with Aggregation-Induced Emission Enhancement Characteristics for Mitochondria-Imaging and Zebrafish-Imaging

Na Li [1], Liyan Liu [1], Huiqing Luo [1], Huaqiao Wang [2], Depo Yang [1] and Feng He [1,*]

[1] School of Pharmaceutical Science, Sun Yat-sen University, Guangzhou 510006, China; lina49@mail2.sysu.edu.cn (N.L.); liuly37@mail2.sysu.edu.cn (L.L.); luohq8@mail2.sysu.edu.cn (H.L.); lssydp@mail.sysu.edu.cn (D.Y.)

[2] Department of Anatomy and Neurobiology, Zhongshan School of Medicine, Sun Yat-sen University, Guangzhou 510006, China; wanghq@mail.sysu.edu.cn

* Correspondence: hefeng@mail.sysu.edu.cn; Tel.: +86-203-994-3036

Table of Contents

^{1}H, ^{13}C NMR spectra and ESI-MS analysis for compound **1** ....Figure S1

^{1}H, ^{13}C NMR spectra and ESI-MS analysis for compound **2** ....Figure S2

^{1}H, ^{13}C NMR spectra and ESI-MS analysis for compound **3**...............................Figure S3

^{1}H, ^{13}C NMR spectra and ESI-MS analysis for compound **4**...............................Figure S4

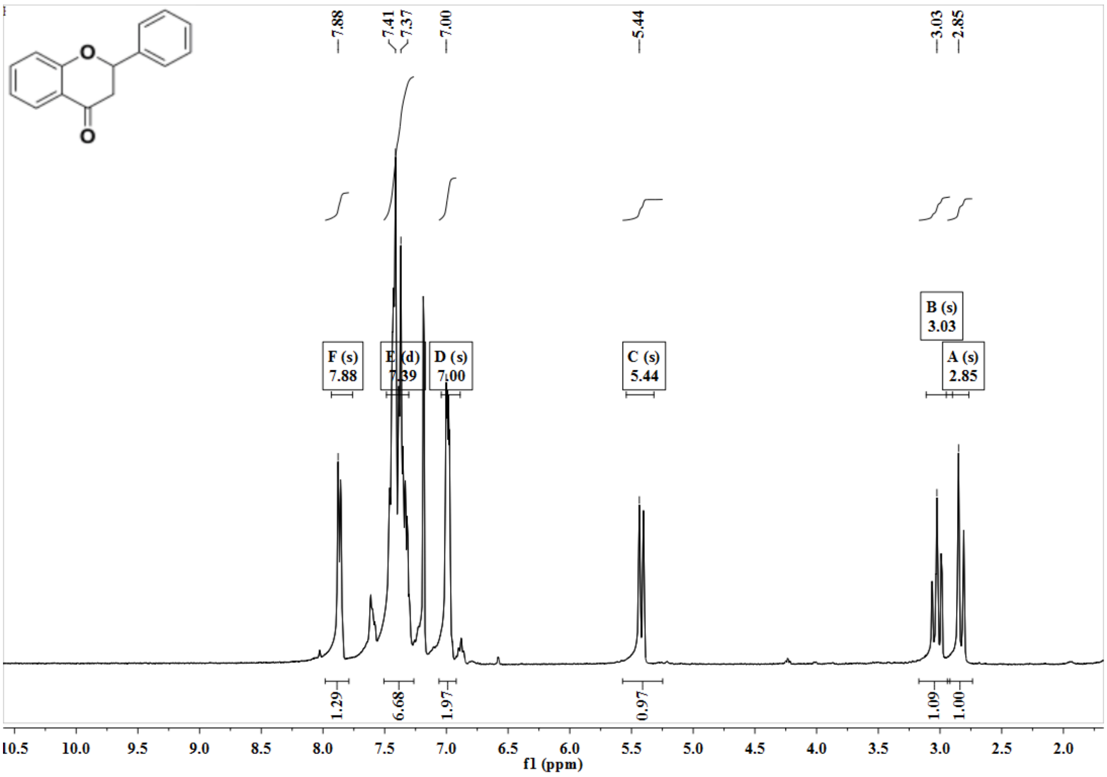
7.88
7.41
7.37
7.00
5.44
3.03
2.85
F (s)
7.88
E (d)
7.39
D (s)
7.00
C (s)
5.44
B (s)
3.03
A (s)
2.85
1.29
6.68
1.97
0.97
1.09
1.00
f1 (ppm)

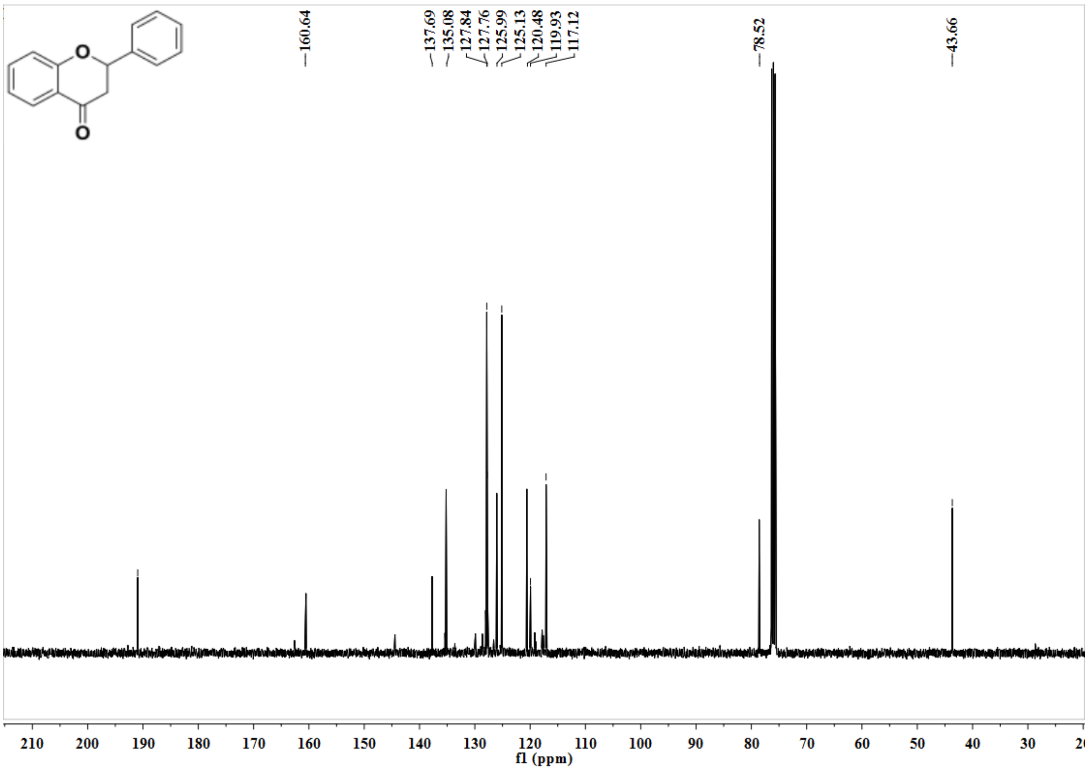
160.64
137.69
135.08
127.84
127.76
125.99
125.13
120.48
119.93
117.12
78.52
43.66
f1 (ppm)

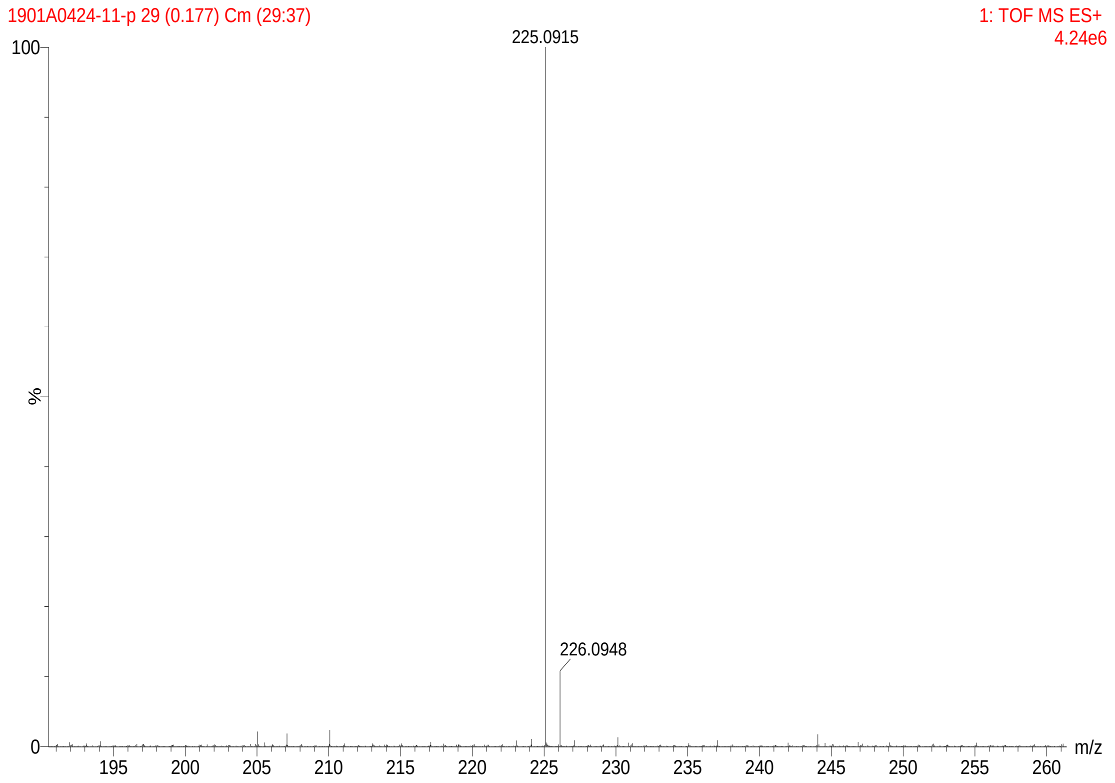

Figure S1. ^{1}H, ^{13}C NMR spectra and ESI-MS analysis for compound **1.**

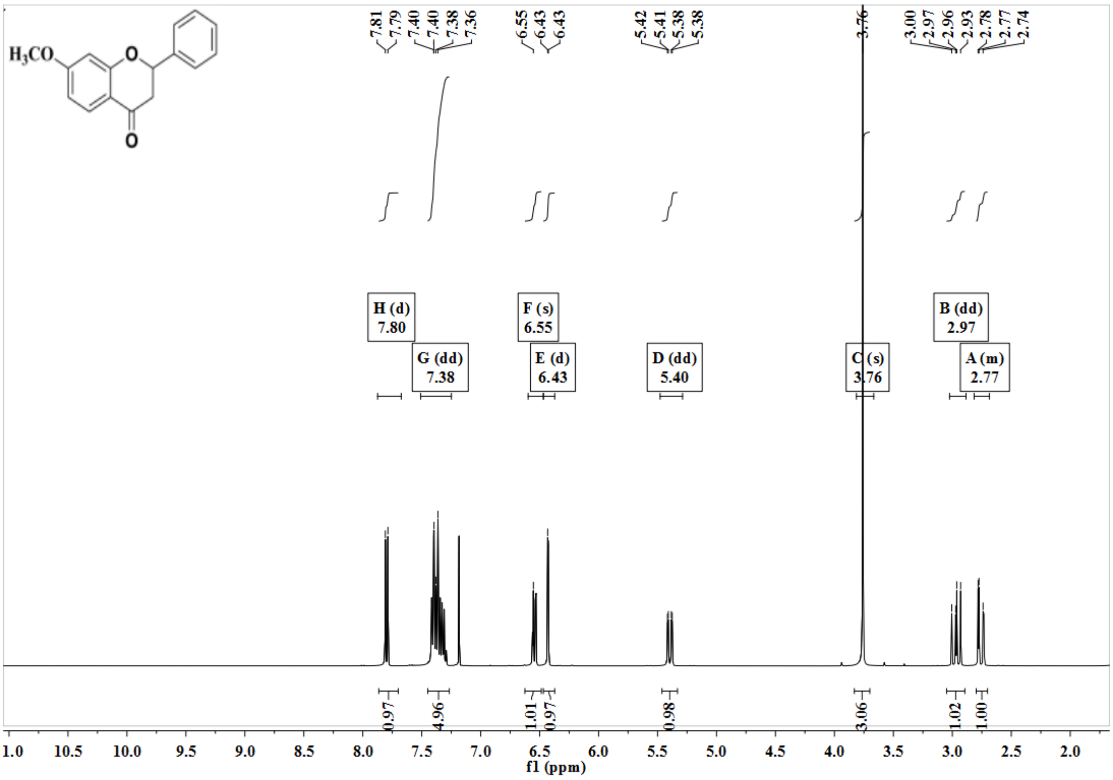
H₃CO
O
O
7.81
7.79
7.40
7.40
7.38
7.36
6.55
6.43
6.43
5.42
5.41
5.38
5.38
3.76
3.00
2.97
2.96
2.93
2.78
2.77
2.74
H (d)
7.80
G (dd)
7.38
F (s)
6.55
E (d)
6.43
D (dd)
5.40
C (s)
3.76
B (dd)
2.97
A (m)
2.77
0.97
4.96
1.01
0.97
0.98
3.06
1.02
1.00
f1 (ppm)

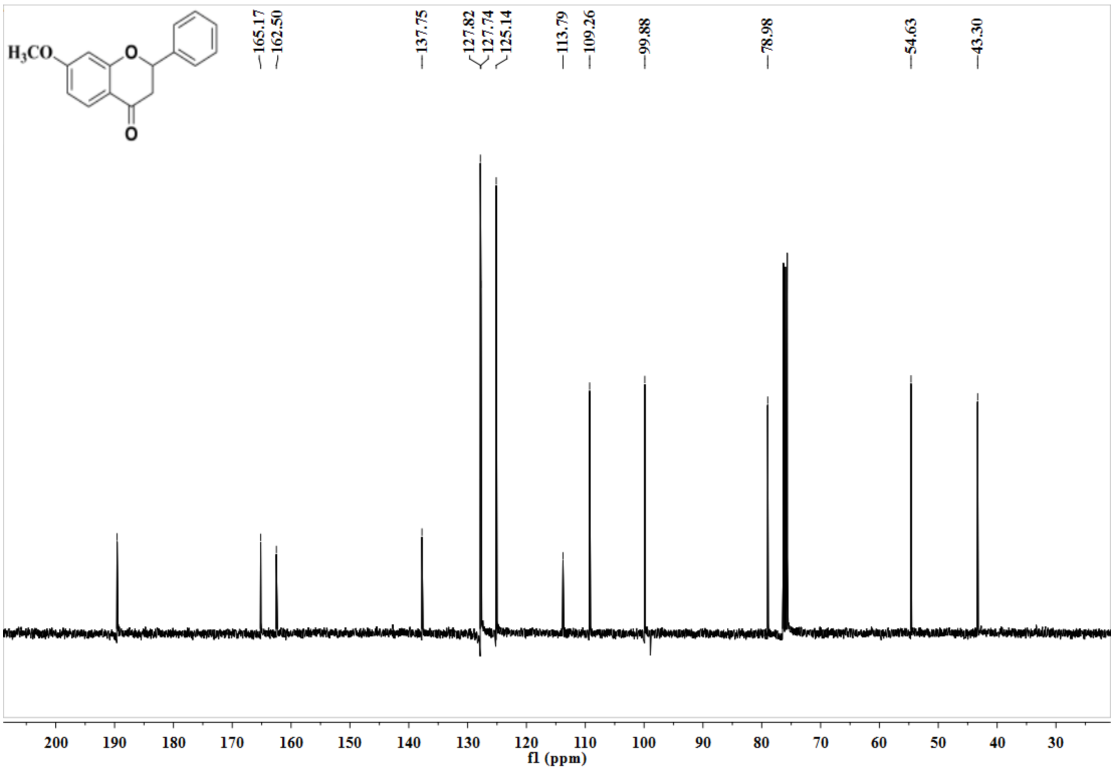
H₃CO
O
O
165.17
162.50
137.75
127.82
127.74
125.14
113.79
109.26
99.88
78.98
54.63
43.30
f1 (ppm)

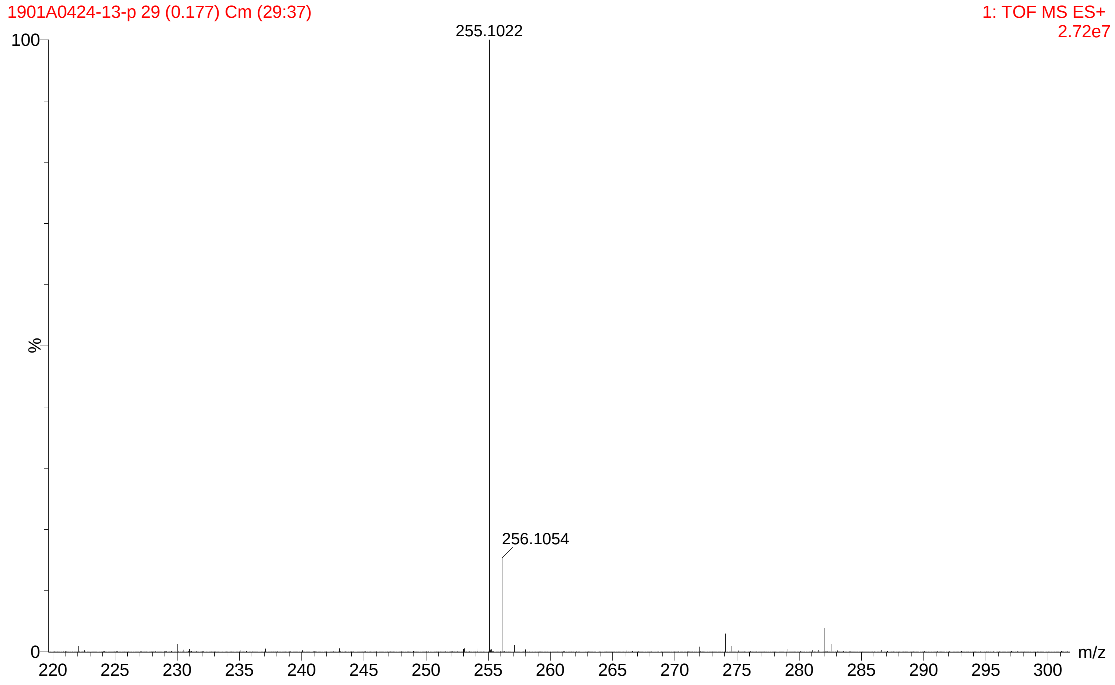

Figure S2. ^{1}H, ^{13}C NMR spectra and ESI-MS analysis for compound **2.**

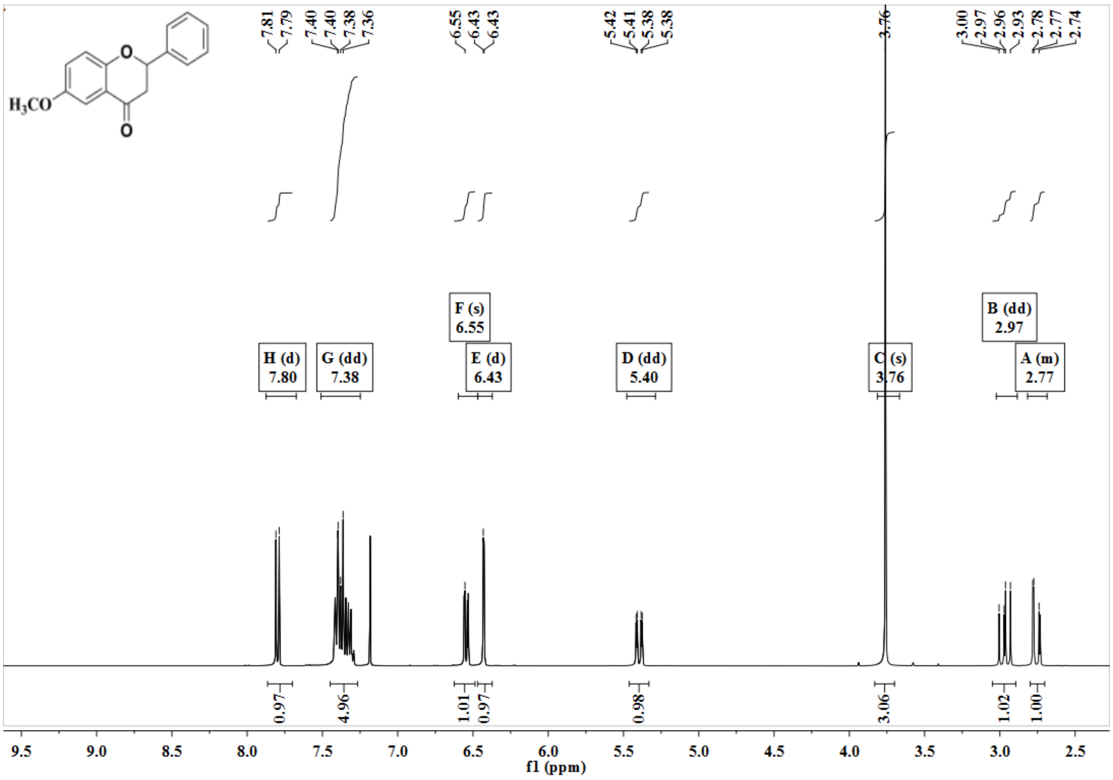
H3CO
O
7.81
7.79
7.40
7.40
7.38
7.36
6.55
6.43
6.43
5.42
5.41
5.38
5.38
3.76
3.00
2.97
2.96
2.93
2.78
2.77
2.74
F (s) 6.55
B (dd) 2.97
H (d) 7.80
G (dd) 7.38
E (d) 6.43
D (dd) 5.40
C (s) 3.76
A (m) 2.77
0.97
4.96
1.01
0.97
0.98
3.06
1.02
1.00
9.5 9.0 8.5 8.0 7.5 7.0 6.5 6.0 5.5 5.0 4.5 4.0 3.5 3.0 2.5
f1 (ppm)

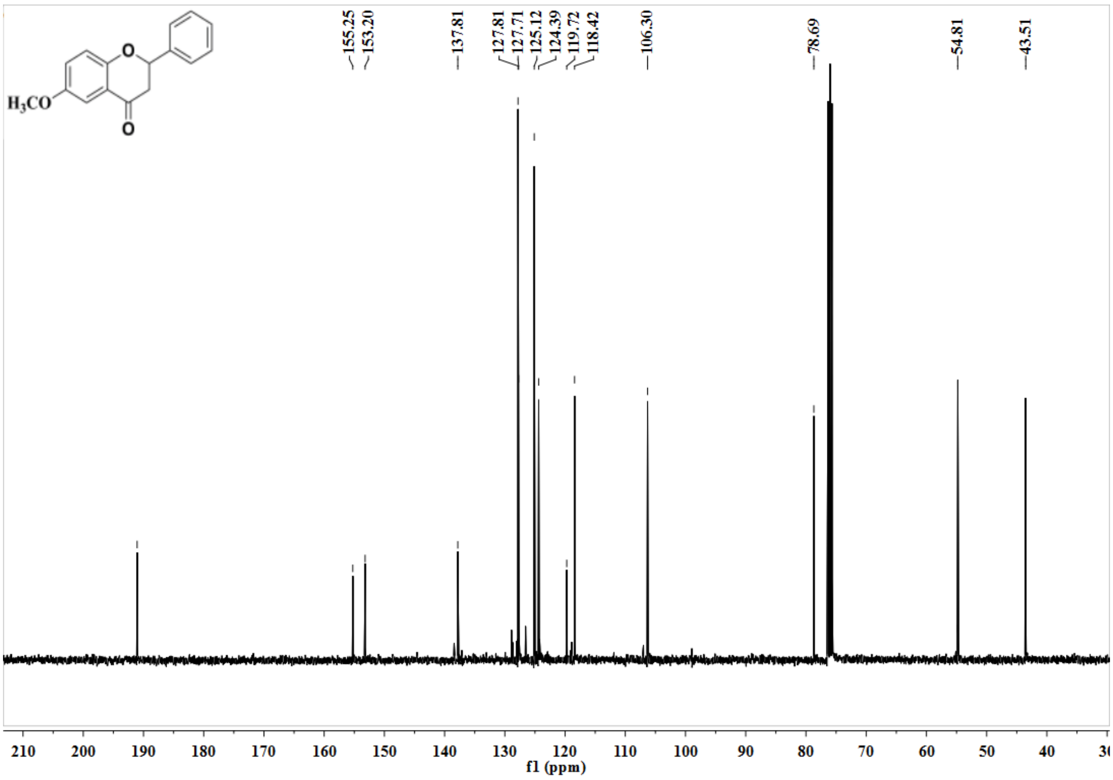
H3CO
O
155.25
153.20
137.81
127.81
127.71
125.12
124.39
119.72
118.42
106.30
78.69
54.81
43.51
210 200 190 180 170 160 150 140 130 120 110 100 90 80 70 60 50 40
f1 (ppm)

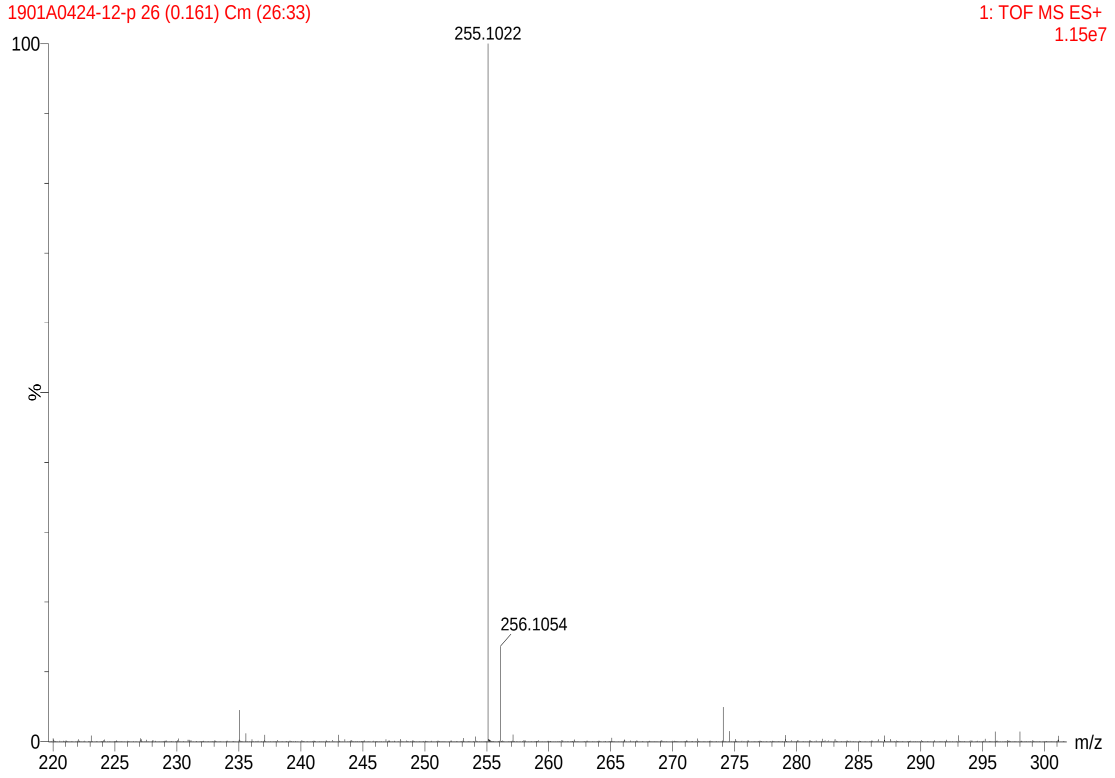

Figure S3. ^{1}H, ^{13}C NMR spectra and ESI-MS analysis for compound **3.**

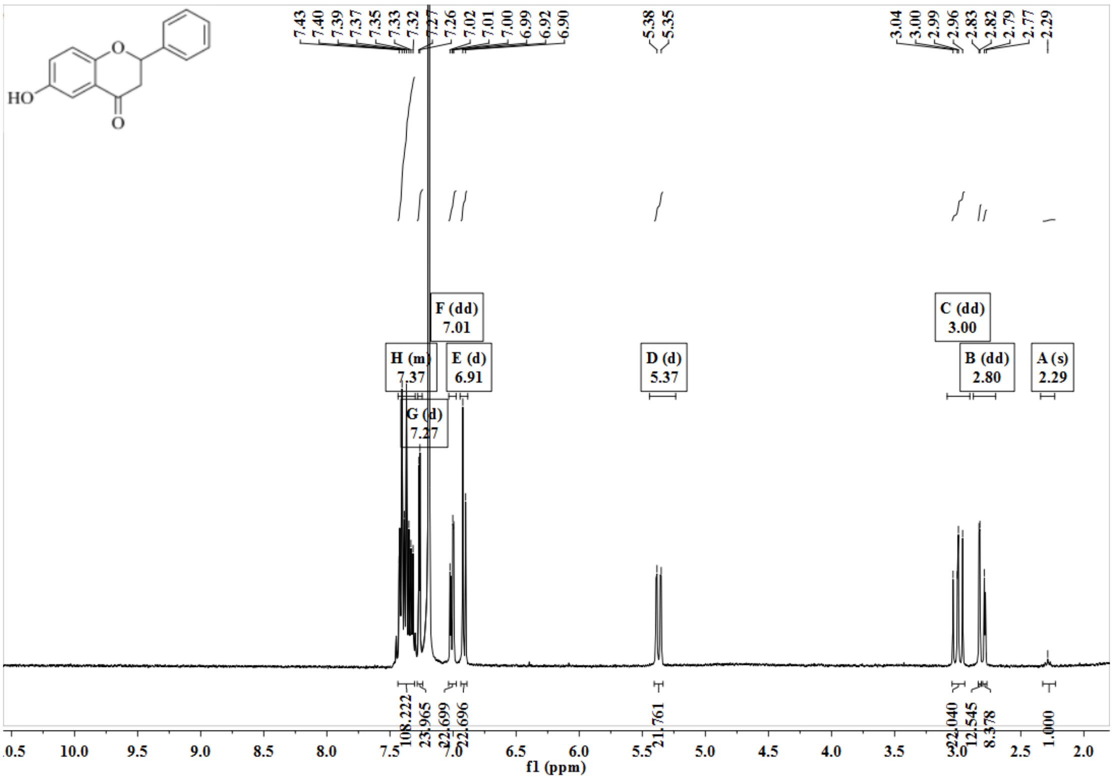

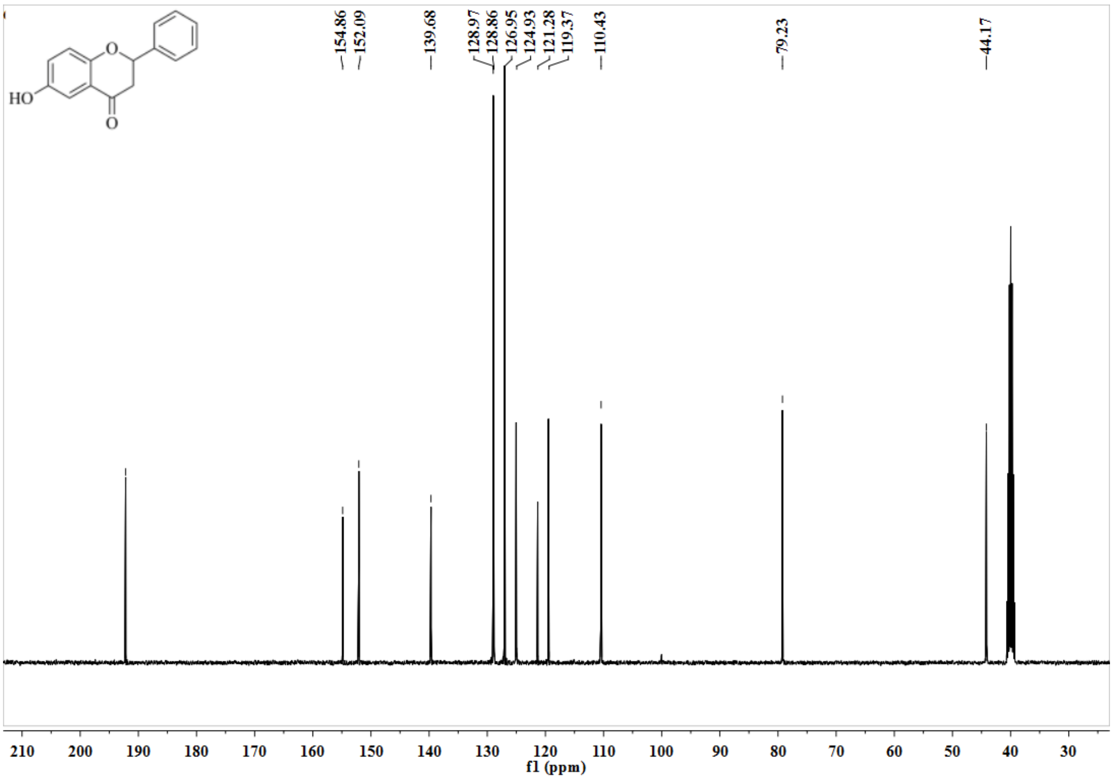

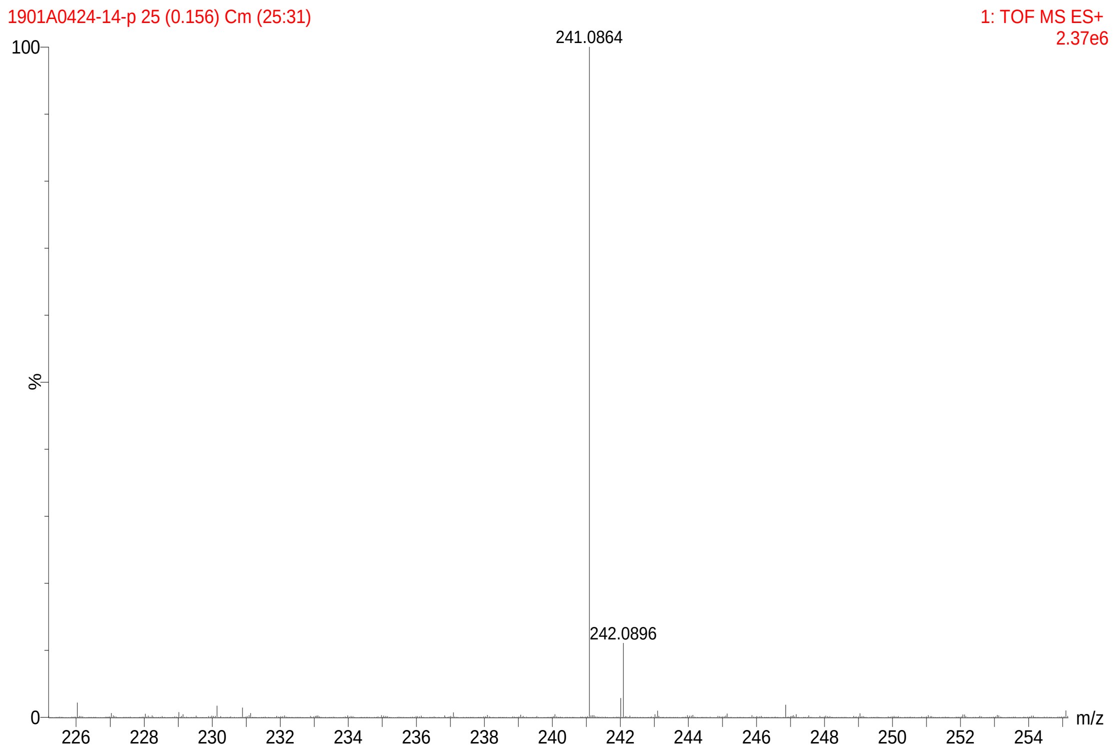

Figure S4. ^{1}H, ^{13}C NMR spectra and ESI-MS analysis for compoud **4**.
